# Measuring continuity of ambulatory cardiovascular care: a cross-sectional study on the applicability of the Nijmegen Continuity Questionnaire in Germany

**DOI:** 10.1186/s12913-022-08612-z

**Published:** 2022-10-18

**Authors:** Patrick Hennrich, Christine Arnold, Jan Koetsenruijter, Michel Wensing

**Affiliations:** Heidelberg University Hospital, Im Neuenheimer Feld 130.3, 69120 Heidelberg, Germany

**Keywords:** Nijmegen Continuity Questionnaire, NCQ, Ambulatory cardiology care, Continuity of care, Germany, General practitioners, Cardiologists, Applicability

## Abstract

**Background:**

Chronic cardiovascular diseases demand continuous care from general practitioners and medical specialists. Especially in fragmented healthcare systems, such as in Germany, a large body of research is devoted to the improvement of care continuity. Meanwhile, measuring continuity of care itself has been a challenge due to the absence of validated instruments. In 2011, the Dutch Nijmegen Continuity Questionnaire (NCQ) was developed and validated to measure continuity of care across care settings from the patients’ perspectives in the Netherlands. Its applicability in other countries and health systems, however, has rarely been tested. We therefore aimed at assessing the applicability of the Nijmegen Continuity Questionnaire to the German health care context.

**Methods:**

We translated and applied the original NCQ to an ambulatory cardiovascular care setting in Germany. Qualitative interview data and quantitative survey data on our adaptation were collected from patients in 27 general practices within the German states of Baden-Wuerttemberg and Rhineland-Palatinate. Qualitative data on understandability and clearness of the questionnaire was obtained using semi-structured telephone interviews and think aloud-protocols. Quantitative data was obtained through an anonymous written questionnaire containing the translated NCQ items to assess applicability of our translation. We performed confirmatory and exploratory factor analyses based on the original NCQ-structure mapped to general practitioners and an aggregated analysis of general practitioners and cardiologists combined.

**Results:**

A total of 6 patients participated in the interviews and a total of 435 patients participated in the written survey. The interviews showed that, overall, patients had little difficulties comprehending and answering to our translation of the NCQ. The confirmatory factor analyses then showed that the structure of the original NCQ with 12 items and 3 latent factors can also be found in the German context. However, a simpler 2-factor-structure would also fit well with the data.

**Conclusion:**

A German translation of the NCQ yielded a factor structure comparable to the original version and proved to be understandable for patients.

**Trial registration:**

The project underlying the study was registered on November 7, 2019 in the German Clinical Trials Register (www.drks.de) under ID: DRKS00019219.

**Supplementary Information:**

The online version contains supplementary material available at 10.1186/s12913-022-08612-z.

## Background

Chronic cardiovascular diseases like coronary heart disease and chronic heart failure are among the main causes of death around the world [1]. They require continuous care of general practitioners and medical specialists alike. However, it can be challenging to achieve this. In Germany, the healthcare system is fragmented [2, 3], not only when it comes to cardiovascular care: General practitioners and medical specialists work in office-based ambulatory practices that are independent from each other. Meanwhile, hospitals mostly provide inpatient and emergency care. Information exchange and care coordination in the ambulatory sector vary, leading to gaps in the continuity of care for patients. There is a large body of research exploring these issues and evaluating interventions to enhance integrated care. However, the measurement of continuity of care itself is hampered by inconsistent definitions and absence of validated measures. Over the years, various instruments have been developed to measure continuity of care from different perspectives and across various areas of health care [4]. Prominent examples include the Bice-Boxerman Continuity of Care Index that focuses on visits, number of care providers involved and referrals [5] or the Usual Provider of Care index that focuses on visits with one’s own health care provider [6]. However, these measures do not necessarily reflect patients’ experiences. In 2011, Uijen et al. developed the Nijmegen Continuity Questionnaire (NCQ), designed to measure continuity of care across care settings from the patients’ perspectives: The 12-item questionnaire proved to be reliable and valid for measuring the continuity of primary care in the Netherlands. Its applicability in other countries and health systems was assumed for countries where the general practitioner plays a gatekeeping role similar to the Netherlands, but it had not been tested [7, 8]. Since then, sporadic and mixed international evidence has been gathered on the applicability of various translations in several settings: A Chinese version of the NCQ in a sample of hypertension patients in a tertiary clinic [9], a Norwegian version in a sample of patients with various conditions in rehabilitation institutions [10] and a Hebrew version in a sample of patients with various cancer types who received oral anticancer therapy in oncology centres [11]. Our study aimed to manufacture a German translation of the NCQ and examine its applicability and usefulness to measure continuity of care in the ambulatory setting in Germany regarding general practitioners and practice-based cardiologists from the patients’ perspectives.

## Methods

### Study design

A mixed-methods observational study was conducted in the German states of Baden-Wuerttemberg (approximately 11 million inhabitants) and Rhineland-Palatinate (approximately 4 million inhabitants). The study was part of the project ExKoCare on the coordination of ambulatory cardiovascular care within general practices and between general practices and cardiologists [12]. The project obtained ethics approval by the Ethikkommission der Medizinischen Fakultät Heidelberg [Ethics Committee of the medical faculty of Heidelberg] under ID S-726/2018. In Baden-Wuerttemberg, an additional approval from the Landesärztekammer (state authorisation association for medical issues) was required and obtained for conducting the patient survey under ID B-F-2020–051. The study was performed in accordance with the Declaration of Helsinki. ExKoCare was registered at the German Clinical Trials Register (www.drks.de) on 07 Nov. 2019 under ID DRKS00019219. The following descriptions adhere to the STROBE Checklist [13] on cross-sectional studies for the quantitative part of the study and the COREQ guidelines [14] for the qualitative part of the study.

### Study sample and recruitment

For the qualitative part of the study we aimed at recruiting a convenience sample of *n* = 10 adult patients with three chronic conditions, at least one of them being a cardiovascular one. Furthermore, they had to be able to participate and complete the study cognitively. Since we expected a response rate of about 50%, documents for a total of 20 suitable patients were prepared. In December 2021, patients were approached in a primary care research practice associated with our department. A total of 12 eligible patients were identified. They were approached by the practice assistant and personally received an envelope containing necessary background information as well as a form where they had to give written informed consent to participate. The practice assistant received a compensation of 150 € for recruiting patients and each patient was offered 30 € as a monetary incentive for participation. Patients who wished to participate had to send the signed written consent form directly to us. Hence, the practice involved in recruitment was unable to find out who actually participated and only knew who they handed the documents out to.

For the quantitative part of the project, we aimed to recruit a total of 600 patients from 40 different general practices, hence we aimed at 15 patients per practice. Here, we also aimed at recruiting patients who suffered from three chronic conditions, at least one of them being a cardiovascular one, preferably coronary heart disease and/or chronic heart failure to ensure a certain complexion that required them to be regularly seen by general practitioners and cardiologists alike. The general practices were participants in the ExKoCare project. The written questionnaires for the ExKoCare project including the NCQ questions and necessary background information were mailed out to the general practices. Each practice was asked to support patient recruitment by creating lists of suitable patients through their practice software and subsequently choose random patients through a varying pattern provided by us via telephone (e.g. “Starting with patient number 5 on the list, contact every third patient”) to avoid introducing selection bias. Afterwards, they had to forward the necessary documents to the respective patients. Since we expected a patient response rate of about 30%, we provided each practice with enough material enabling them to mail out a maximum of 50 questionnaires. Questionnaires were mailed out to the patients between October 2020 and October 2021, depending on when the respective practice joined the ExKoCare project. When patients sent back the anonymous questionnaire, it was interpreted as consent since these patients were not involved in the study in any other way. Additional declarations of consent were waived by the Ethics Committee of the medical faculty of Heidelberg since we used blinding measurements so the practices were unable find out which of their patients responded while we were unable to track which patients were invited and the quantitative study was therefore anonymous. Similar to the qualitative phase, the practices only knew who they sent a questionnaire to. We could only link each incoming questionnaire to the respective practice through an ID, but not to the patients themselves. All practices and patients were informed about this in writing, as well as about the fact that returning the questionnaire was understood as consent.

### Measurements and data-collection

The English version of all 12 items in the NCQ was translated carefully by both CA and PH using a forward translation into German and a subsequent backwards translation into English. Both researchers manufactured their forward- and backwards translation separate from each other. After each translation, both versions were compared and possible differences were discussed until consensus was reached. The process was supervised by MW who knows general practice in Germany and the Netherlands well and is proficient in English, German and Dutch. Our translated version of the NCQ can be found in Additional file 1. For the qualitative part of the study, we conducted semi-structured telephone-interviews. The interview guideline included our translation of the NCQ as well as two open-ended questions afterwards. Interviews were planned to roughly last 15–30 min. All interviews were conducted by PH who gathered experience with qualitative interviews over more than four years in various studies in the field of health services research. Patients had no prior relationship to the researcher except that they knew about him being a member of the research team because of the invitation letter to the study. During the interview, each NCQ-question and answer was read out similar to a computer assisted telephone interview. Patients were asked to answer the questions and talk about their thoughts and any noticeable aspects they faced during the interview, so we were able to make a think aloud-protocol. During the interview, patients were at least once reminded of thinking aloud. The two open-ended questions that followed the NCQ-questions dealt with possible difficulties regarding answering and understanding the NCQ-questions. Interviews were audio-recorded and no repeated interviews were conducted.

The quantitative part of the study consisted of a written questionnaire that included our German translation of the NCQ. In the original study by Uijen et al. [7, 8], each of the 12 NCQ items on personal continuity and team/cross boundary continuity had to be answered twice: Once in regard to each patient’s general practitioner and once in regard to the medical specialist. Additionally, in the original study, the four items on cross boundary continuity were surveyed regarding general practitioners and medical specialists together.

The project underlying our study focused primarily on cardiovascular care provided by general practitioners and we aimed at evaluating the applicability of the NCQ across the ambulatory cardiology sector as a whole. Therefore, we decided to slightly adapt the original approach: Like in the original study, all 12 items were surveyed regarding general practitioners. Then, instead of repeating this for cardiologists, we only surveyed the eight items on personal continuity regarding cardiologists. The additional four items on cross boundary continuity were surveyed regarding general practitioners and cardiologists together and then combined with a mean of the personal continuity items across general practitioners and cardiologists to allow for an aggregated analysis of cardiovascular ambulatory care.

In the original NCQ, primary care provider and specialist had to be the most important one from each patient’s perspective. In Germany, patients often have “their” GP, meaning they are seen by the same physician every time they receive primary care – hence this GP can be assumed to be the most important primary care provider. Regarding specialists, due to the aims of the ExKoCare-project, we decided to fix the provider as being the cardiologist to ensure the desired focus on cardiovascular care. Typically, if patients see a cardiovascular specialist in Germany, this is a cardiologist. However, we cannot ensure that the cardiologist is always perceived as the most important specialist by patients.

As described above, data were collected anonymously through the written questionnaires. Patients were asked to answer each of the items on a Likert-scale from 1 (“Fully agree”) to 5 (“Do not agree at all”). They were also given the option to answer “I don’t know/not sure” for each item. To ensure that answers represented the patients’ current opinions, questions only referred to physician appointments during the past 12 months. Patients that had not seen their GP or specialist during the past year were asked to skip the questions regarding the respective physician.

### Data-analysis

The interviews were transcribed verbatim. However, since the actual answers to the NCQ were not the focus here, the transcripts were then analysed only regarding the think aloud protocol and the two questions at the end of the interview guideline. There were no specific themes or categories defined in advance to ensure openness to any issues regarding the NCQ that may arise. All transcripts and protocols were carefully checked by PH regarding aspects that affect understandability and application of the translated NCQ to German patients. Transcripts were not provided to the patients for additional comments.

The filled-out questionnaires were scanned, missing and ambiguous answers were coded respectively and a dataset was created. The coding of the NCQ-questions was reversed so that 1 now indicated the category “Do not agree at all” and 5 indicated the category “Fully agree”. Cases were only included in the factor analyses when there was no missing value on the respective items of the NCQ and the “I don’t know/not sure”-option was not used. This led to three subsamples in addition to the total sample: The sample for the confirmatory factor analysis (CFA) using NCQ-data on general practitioners, the descriptive sample using NCQ-data on personal continuity among cardiologists and the sample for the CFA using aggregated NCQ-data on both GPs and cardiologists. Sociodemographic data of participants were compared between the total sample and all three subsamples using T-tests and Chi^2^-Tests. Respondents’ answers to all NCQ-items were then analysed descriptively using means, standard deviations (SD) as well as absolute and relative frequencies. For the CFA, we defined factors according to the original paper by Uijen et al. [7] with the 12 items loading on a total of 3 latent factors, resulting in the structure shown in Table 1. Model fit was assessed using Comparative Fit Index (CFI), Tucker-Lewis-Index (TLI), Root Mean Square Error of Approximation (RMSEA) and Standardized Root Mean Square Residual (SRMR). We used cut-off values as defined by Hu & Bentler, where a good fit is indicated by CFI and TLI > 0.9, a RMSEA < 0.06 and an SRMR < 0.08 [15]. Additionally, an explorative factor analysis (EFA) was performed to test whether other solutions might also fit to the data. All quantitative analyses were carried out using R 4.1.0 [16].

**Table 1:** Factors and items in the Nijmegen Continuity Questionnaire, adapted from Uijen et al. (2011)

Factor number	Factor name	Item number	Item name
1	Personal continuity: Care provider knows me	1	I know my general practitioner/this cardiologist very well
		2	My general practitioner/this cardiologist knows my medical history very well
		3	My general practitioner/this cardiologist always knows very well what he/she did previously
		4	My general practitioner/this cardiologist knows my familial circumstances very well
		5	My general practitioner/this cardiologist knows my daily activities very well
2	Personal continuity: Care provider shows commitment	6	My general practitioner/this cardiologist contacts me if it is needed, I do not have to ask
		7	My general practitioner/this cardiologist knows very well what I believe is important in my care
		8	My general practitioner/this cardiologist keeps in contact sufficiently when I see other care providers
3	Team and cross-boundary continuity	9	These care providers transfer information very well to each other
		10	These care providers work together very well
		11	The care of these care providers is very well connected
		12	These care providers always know very well from each other what they do

## Results

### Qualitative analyses

Out of the 12 patients approached, a total of 8 patients returned the necessary documents and gave written consent to participate in an interview. Two of these patients later withdrew their consent due to ongoing health issues which made them unavailable for an interview. Both did not agree to postpone the interview to a later date, so a total of 6 patients (4 female and 2 male) eventually participated in an interview. Interviews lasted between (roughly) 5 and 20 min and were conducted between December 2021 and January 2022. Regarding the setting of the interviews, from what we were able to see, patients participated from their home. In one case there was a non-participant present as well before the interview (spouse of the participant) – however, we did not learn whether the person remained in the room during the interview itself.

Overall, patients felt that the NCQ translation was easy to understand and that they were able to answer each question without noticeable difficulties.

Patient 1: *“As a layman, I imagined it to be more complicated, but…yeah, it went well.*”

Patient 4: *“What I liked was that there were no latin words or some kind of technical terms. I think that’s important…because then you’d have to think but don’t want to make a fool of yourself by asking: ‘Excuse me, but I don’t know what that means’.”*

Patient 6: *“No, there was nothing obtuse.*”

Still, in one case, the term “care provider” (German:”Leistungserbringer”) was perceived as confusing:

Patient 4: *“Yes, when it comes to the care provider, that confused me. […] Cooperation of care providers in the family practice…for me, that’s not clearly defined. I would prefer ‘cooperation within the practice’. With ‘care provider’ you don’t know who that is […] But besides that, I understood everything quite well – this was the only aspect that confused me a little.”*

Additionally, in one case, the ability to answer the question on cooperation between care providers in the general practice was limited at first:

Patient 4: *“I can’t say that objectively. I’d say ‘neutral’, I don’t have the insight into how this thing is working.”*

This was resolved after it was explained to the patient once more that the subjective impression is important and that they do not need to assess cooperation in an objective way.

Furthermore, the question on care being well connected was interpreted by the patients in different ways—one being more focused on communication between the care providers and one being more focused on provision of equally suitable care regardless of the individual provider within the practice:

Patient 2: *“[That] they talk to each other, I can have a practice assistant on the phone and they call me back every time […] they are able to assess me well.”*

Patient 3: *“That, when I enter the practice, that the assistants know what’s going on when they look into my file […] that, no matter which assistant is there, I am well cared for.”*

Patient 4: *“Well, that I am cared for well, and cared for correctly by the physician and the physician’s assistant.”*

Patient 5: *“That communication between physician and physician’s assistant works.”*

Patient 6: *”That everything is discussed and passed on.”*

### Quantitative analyses

A total of 28 practices agreed to participate in recruiting patients, of which 26 practices eventually sent out the written questionnaires of the ExKoCare-project including the translated version of the NCQ. Questionnaires were mailed out to a total of 811 patients. Four of the practices had fewer than the desired 15 patients who were eligible for the study. A total of 435 patients (53.64 %) returned the questionnaire. Out of the 435 patients, 287 patients answered all questions in the NCQ regarding their general practitioner, 272 patients answered all questions regarding personal continuity in the cardiology practice and 210 patients answered all questions regarding both. All other patients had at least one missing value or checked the “I don’t know/not sure”-option at least once on the respective part of the NCQ. Table 2 shows basic, sociodemographic characteristics of the total sample and the subsample that was included in the confirmatory factor analyses on the NCQ regarding general practitioners.

**Table 2:** Sociodemographic characteristics of patients participating in the quantitative study

Characteristic	Patients, total sample (*n* = 435)	Patients, NCQ-subsample regarding general practitioners (*n* = 287)
Age (mean (sd)) [n]	76 (8.95) [406]	75 (9.04) [269]**
Sex (n(%))		
*Male*	316 (72.97)	212 (73.87)
*Female*	117 (27.02)	73 (25.44)
Employment (n(%))		
*Not employed (retired, student, unemployed)*	382 (89.67)	250 (87.11)
*Full-time employment (*> *35 h/week)*	34 (7.98)	24 (8.36)
*Part-time employment (*< *35 h/week)*	10 (2.35)	7 (2.44)
Health insurance (n(%))		
*Statutory health insurance*	380 (89.20)	253 (88.15)
*Private health insurance*	39 (9.15)	21 (7.32)
*Self-pay patient*	4 (0.94)	2 (0.70)
*Other*	3 (0.70)	2 (0.70)
Cardiovascular disease for which the patient currently receives treatment (n(%))		
*Hypertension*	281 (64.59)	195 (67.94)
*Cardiac arrhythmia/atrial fibrillation*	130 (29.89)	87 (30.31)
*Coronary heart disease*	244 (56.09)	174 (60.63)*
*Chronic heart failure*	67 (15.40)	50 (17.42)
*Stroke*	40 (9.43)	27 (9.41)
*Peripheral vascular disease*	41 (8.74)	24 (8.36)
*Aortic aneurysm*	17 (3.91)	9 (3.14)
*Other*	36 (8.27)	24 (8.36)
Participation in general practitioner-centred care (n(%)) [n]	204 (55.7)	142 (57.49) [247]
Participation in a disease management programme (n(%)) [n]	204 (53.13)	149 (57.31)* [260]

^*^p < 0.05; **p < 0.01

As the table shows, the subsample is similar to the total sample regarding sociodemographic aspects. Significant differences were only found regarding age (two-tailed t-test, t (404) = -3.21 [95 %-CI: -4.81; -1.15], *p* = 0.001), the presence of coronary heart disease (Chi^2^(1) = 6.51, *p* = 0.01) and whether the patients participated in a disease management programme (Chi^2^(1) = 5.15, *p* = 0.02). Sociodemographic data on the two other subsamples included in the CFAs regarding cardiologists as well as GPs & cardiologists combined can be found in Additional file 2. These samples were similar to the total sample as well and significant differences occurred only regarding age, the presence of coronary heart disease, and the presence of chronic heart failure.

Subsequently, we performed a descriptive analysis of the items included in the NCQ, separated by the respective physician group and in total. The aggregated results on personal continuity are mean values from each patient who answered the respective question for both groups of physicians. The results are shown in Table 3. The numbers of respondents differ from Table 2 because, as explained above, respondents in the factor analyses were not allowed to have missing values on any of the respective variables. A detailed breakdown of the various types of missing values per item can be found in Additional file 3.

**Table 3:** Descriptive results on all translated NCQ-factors (separated by groups of physicians and in total)

**Item number**	**Item name**	**Result regarding general practitioner**^a^ **(mean(sd) [n])**	**Result regarding cardiologist (mean(sd) [n])**	**Aggregated result regarding general practitioner & cardiologist**^b^ **(mean(sd) [n])**
1	I know my general practitioner/this cardiologist very well	4.25 (0.75) [426]	3.74 (0.91) [338]	4.00 (0.68) [333]
2	My general practitioner/this cardiologist knows my medical history very well	4.44 (0.64) [420]	3.97 (0.86) [335]	4.21 (0.62) [325]
3	My general practitioner/this cardiologist always knows very well what he/she did previously	4.15 (0.78) [410]	3.82 (0.91) [322]	4.01 (0.68) [310]
4	My general practitioner/this cardiologist knows my familial circumstances very well	3.98 (0.95) [415]	2.91 (0.97) [320]	3.46 (0.75) [313]
5	My general practitioner/this cardiologist knows my daily activities very well	3.53 (0.95) [394]	2.94 (0.95) [324]	3.26 (0.80) [302]
6	My general practitioner/this cardiologist contacts me if it is needed, I do not have to ask	3.81 (1.00) [414]	3.06 (1.12) [321]	3.46 (0.83) [312]
7	My general practitioner/this cardiologist knows very well what I believe is important in my care	4.01 (0.78) [410]	3.45 (0.99) [318]	3.75 (0.74) [305]
8	My general practitioner/this cardiologist keeps in contact sufficiently when I see other care providers	3.80 (0.92) [407]	3.05 (1.07) [306]	3.44 (0.82) [297]
9	These care providers transfer information very well to each other	4.14 (0.70) [359]	n.a	4.01 (0.72) [318]
10	These care providers work together very well	4.11 (0.74) [352]	n.a	3.81 (0.85) [311]
11	The care of these care providers is very well connected	4.03 (0.76) [347]	n.a	3.74 (0.87) [305]
12	These care providers always know very well from each other what they do	3.81 (0.85) [341]	n.a	3.66 (0.92) [296]

^a^Items 9–12 on cooperation refer to cooperation between providers within the general practice

^b^Items 9–12 on cooperation refer to cooperation between the general practitioner and the cardiologist

As the results show, patients gave the items on continuity of care in the general practice a rating between 3.53 and 4.44 on the Likert-scale. The data regarding GPs showed a considerable ceiling effect on all items, with 16.75% - 50.48% of respondents choosing the highest possible category. Meanwhile, continuity of care in the cardiology practice was rated between 2.91 and 3.97, indicating a possible tendency towards the central category on the scale. Here, we found ceiling effects on the first 3 items where 21.30% - 26.87% of respondents chose the highest possible category. Finally, we performed confirmatory factor analyses for general practitioners separately as well as a combined analysis including both groups. As we already mentioned in the methods section, the model partly replicated the original NCQ-paper [7] and included only cases where each item on the respective scale (GP, cardiologist, total) was answered. The two confirmatory factor analyses led to the factor loadings and standard errors (se) shown in Table 4, factor variance was set to 1.

**Table 4:** Results of the confirmatory factor analysis on the translated NCQ (separated by general practitioners and in total)

Factor number	Item number	General practitioners only (*n* = 287)	General practitioners & Cardiologists (*n* = 210)
		*Loading (se)*	*Loading (se)*
1	1	0.56 (0.04)	0.45 (0.04)
	2	0.54 (0.03)	0.42 (0.03)
	3	0.61 (0.04)	0.48 (0.04)
	4	0.73 (0.05)	0.61 (0.05)
	5	0.65 (0.05)	0.57 (0.05)
2	6	0.75 (0.05)	0.61 (0.05)
	7	0.66 (0.04)	0.60 (0.04)
	8	0.78 (0.05)	0.71 (0.05)
3	9	0.63 (0.03)	0.64 (0.04)
	10	0.71 (0.03)	0.80 (0.04)
	11	0.72 (0.03)	0.83 (0.04)
	12	0.71 (0.04)	0.78 (0.04)

**Fig. 1:**
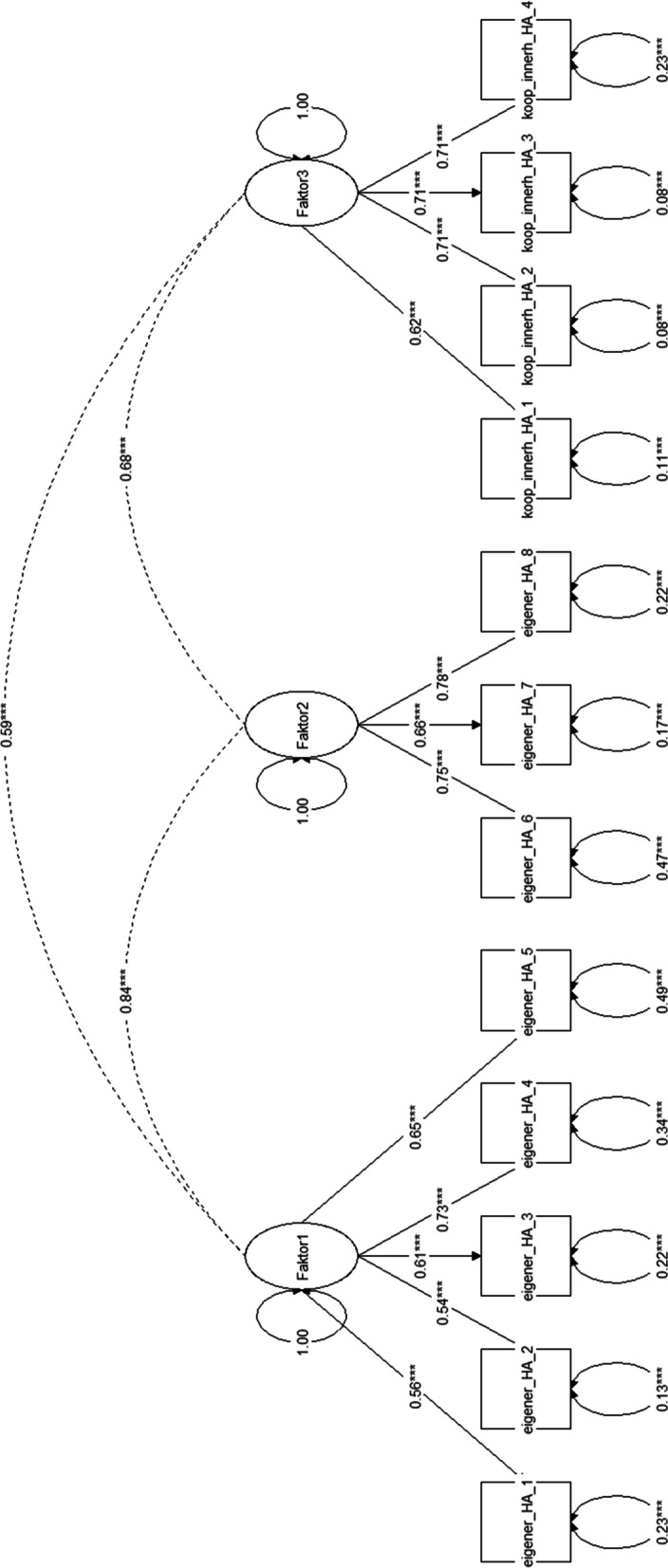
Structural equation model for the translated NCQ on general practitioners. Legend/translations Fig. 1: “Faktor1-3” = ”Factor 1-3”; “eigener_HA_1-8″ = items 1 – 8 on personal continuity within the general practice; “koop_innerh_HA_1 – 4″ = items 9 – 12 on team continuity within the general practice

Correlation between factors varied, with an overall high correlation between both of the “personal continuity”-factors: The general practitioner model (Fig. 1) showed a correlation of *r* = 0.84 between factors 1 and 2, *r* = 0.68 between factors 2 and 3 and *r* = 0.59 between factors 1 and 3. The model had a CFI of 0.95, a TLI of 0.94, an RMSEA of 0.09 and an SRMR of 0.04. While CFI and TLI both indicated a good fit, the RMSEA of 0.09 was on the upper threshold of being categorized as acceptable fit, since everything above 0.1 is deemed poor [15]. Meanwhile, the SRMR indicated a good fit. Overall, judging by the indicators, the model fit can be categorized as acceptable.

**Fig. 2:**
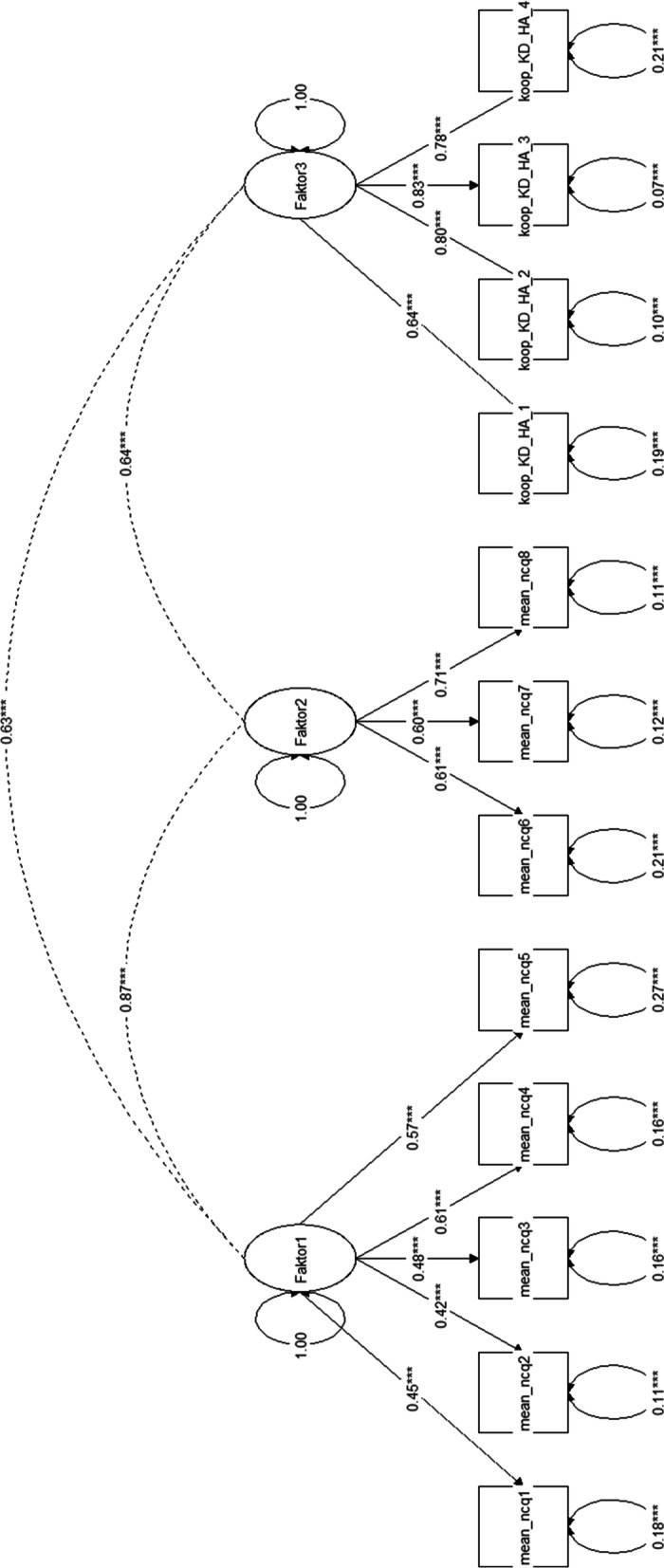
Structural equation model for the translated NCQ on general practitioners and cardiologists combined. Legend/translations Fig. 2: “Faktor1-3” = ”Factor 1-3”; “mean_ncq1-8″ = mean of items 1 – 8 across GPs and cardiologists; “koop_KD-HA1 – 4″ = items 9 – 12 on cross boundary continuity between GPs and cardiologists

The combined model that encompassed GPs and cardiologists alike (Fig. 2) showed correlations of *r* = 0.87 between factors 1 and 2, *r* = 0.64 between factors 2 and 3 and *r* = 0.63 between factors 1 and 3. Model fit indicators showed a CFI of 0.94, a TLI of 0.93, an RMSEA of 0.11 and a SRMR of 0.04. Hence, all indicators besides the RMSEA indicated a rather good fit. Overall, this again can be seen as an acceptable fit.

Since the CFA indicated a high correlation > 0.8 between the latent “personal continuity”-factors 1 and 2 in both models, we decided to perform a coarse exploratory factor analysis (EFA) using the data of the third model that combined GPs and cardiologists. The results supported the correlation coefficients found in the CFA: The EFA allowed for three factors with high loadings (cut-off was set at 0.5) for items 1–3 on factor 3, for items 4–8 on factor 1 and for items 9–12 on factor 2. A simpler structure that set the data to two factors was possible as well without causing insignificant items. The two-factor model showed high factor loadings (cut-off was set at 0.5) for items 1–8 on factor 1 and for items 9–12 on factor 2.

## Discussion

The Nijmegen Continuity Questionnaire is a valuable tool to measure continuity of care from patients’ perspectives. Its applicability in the German ambulatory context had not yet been tested. Our study applied the Dutch NCQ in an ambulatory, cardiovascular care setting in two German states. We performed qualitative telephone interviews and a confirmatory factor analysis with our empirical data and the specifications of the NCQ as described in the original authors’ paper [7, 8]. The translated NCQ proved to be understandable for patients and replicated the three domains found in the original version, but a two domains approach seemed to fit well with the data structure too.

Quantitative results indicated that the factors and distribution of items in the original NCQ [7, 8] can be found in the German context as well. While there was no strong evidence against applicability of the original NCQ to our data, the model fit of the adaptation varied between the two settings we explored and showed room for some additional modifications. Even though the Dutch and German health systems have some aspects in common, they are not identical, so this should not be surprising – the original model is not completely tailored to the German context. Furthermore, it has to be noted that, in recent years, the used cut-off values to estimate model fit introduced by Hu and Bentler have been disputed for their inflexibility [17]. Hence, we cannot rule out that the applied cut-off values might simply have been too strict and therefore indicated room for improvement.

Especially the personal continuity-factors “care provider knows me” and “care provider shows commitment” were highly correlated with *r* = 0.84 and *r* = 0.87 and our exploratory factor analysis indicated that these latent constructs largely described the same concept and could also be represented by one factor instead of two. However, from a theoretical point of view, it is questionable whether a two-factor NCQ would bear any additional value besides being slightly more efficient in its structure: The original NCQ showed similar results with a high correlation between these two factors of *r* = 0.61 and the authors argued that knowing a patient is a prerequisite for showing commitment [7]. That the correlation was even higher in our study, in our opinion, does not falsify this assumption and hence we see no clear indication to resort to a two-factor NCQ for future applications. In the German context, knowing a patient before might simply be even more connected to showing commitment. This may result from how ambulatory cardiovascular care is organized in Germany: Personal continuity of care has been shown to be quite high compared to other countries [18]. Ambulatory cardiovascular care is usually provided by GPs and cardiologists. When it comes to patients with known conditions and ongoing medication, the medical specialist is typically involved once or twice a year for routine check-ups. The remaining time, patients are seen and treated by their respective GP. When they participate in a structured programme, such as a DMP, they see their GP four times a year regarding their cardiovascular condition alone. Additionally, for other medical issues and conditions that do not require emergency services, patients in Germany usually at first see their GP as well, even when the GP eventually refers them to a medical specialist. When it comes to specialists, over time patients often stick to seeing one particular medical specialist in the respective field. These circumstances might lead to a very strong sense of knowing each other and mutual trust regarding one’s own GP and specialists. This is not necessarily present in other health systems where, e.g., patients visit outpatient clinics where they do not have “their” physician but can be seen by any physician that is currently on duty.

What meets the eye is that, looking at the initial descriptive analysis, patients rated each of the 12 items worse or more neutral when the questions referred to their cardiologist and the respective questions were completed by fewer respondents. As it turned out from other questions and as we described above for the German health care system, a substantial number of patients was mainly treated by their GP and rarely saw a cardiologist, despite the cardiovascular nature of their condition. One can assume that these patients did not know their cardiologists as well as patients who saw them more regularly. These circumstances might have led to a neutral position towards the cardiologists and more missing values. On the other hand, answers being close the central category 3 on a 1–5-scale might indicate possible biases. Here, a central tendency error could have been introduced: When Likert-scales offer a midpoint (like ours did), respondents might generally tend to choose this central category of a scale [19]. This would, however, not explain why the central tendency was only present for cardiologists and not for GPs. A more convincing explanation would be that the questions on cardiologists were asked after those on GPs, which probably led to satisficing effects: It is known that, due to increasing cognitive effort, the longer a questionnaire is, the more respondents might choose simple or acceptable categories on scales, such as central or neutral ones [20]. Regarding the number of missing values, there might have simply been a methodology bias due to the ongoing COVID-19 pandemic: As mentioned in the methods section, patients were asked to indicate/rate contacts with their physicians in case they had an appointment during the past 12 months. Several practices postponed non-urgent appointments during the pandemic so that patients who only see a medical specialist for routine check-ups probably simply did not see one during the past year. In light of these potential biases, the descriptive results on cardiologists should not be overinterpreted here.

Qualitative results showed that our translation of the NCQ was overall easy to understand and caused only minor challenges for the respondents. The definition of “care provider” was unclear in one case. This is an important aspect, since this rather technical term is widespread in Germany – however, mainly in a scientific or legal environment. A patient who is not familiar with these domains might have a need for clarification here. In future applications, the word “care provider” therefore could be replaced by more common occupation-related terms like “physician” and “physician’s assistant”. On the other hand, this would limit the question to a specific occupation, while the current version allows for a broader approach. Additionally, in our questionnaire it was clearly stated in an introductory sentence who the questions refer to.

The term “well connected” care in the family practice turned out to be rather vague. It was understood by respondents in various ways: One more focused on communication between care providers and one more focused on equal competences of care providers. In some cases, this was particularly understood as a term regarding the knowledge and abilities of practice assistants. This ambiguity might result from the item generation process of the original NCQ, which was based on extensive literature research and then tailored to the Dutch context and not the German setting [7]. The respective item is part of the latent “team and cross-boundary continuity” factor, which contains both items on knowing about procedures that have been performed and items on communicating with other care providers. These concepts are comparable to the interpretation by the respondents in our study, so it seems unlikely that the ambiguity of the term actually leads to a problem when looking at the NCQ as a whole.

### Strengths and limitations

Our study tested the applicability of the Nijmegen Continuity Questionnaire in Germany. To our knowledge, this has not been done before, even though the NCQ was developed in a different health care context and evidence on whether it is suited for other contexts is still rather scarce. Hence, the work adds to the knowledge on international applicability of survey instruments outside of their country of origin.

The main weakness can be found in the low number of participants in the qualitative study. Since the interviews yielded highly homogeneous results from the beginning, we felt that saturation had been reached and did not recruit further participants. Still, even though the number of participants is not the decisive aspect in a qualitative study, 6 participants do not allow us to confidently assure that all potential issues regarding the NCQ have been sufficiently covered.

Furthermore, the qualitative study had to be conducted via telephone. This might have led to incomplete data collection, as especially non-verbal reactions to questions could not be recorded. Usually, when applying the “thinking aloud”-approach, participants are presented with the written questionnaire which they then fill out while thinking aloud. However, due to the ongoing COVID-19-pandemic, personally visiting patients was not possible. Alternative approaches, such as presenting the questionnaire via internet, were discarded because, due to the typically advanced age of participants, we could not rule out major difficulties in handling online questionnaires or a video call program and patients withdrawing from the study as a result.

The ceiling effects observed in the quantitative study, especially on questions regarding the patients’ GPs, furthermore might hint at social desirability effects. Since the questionnaires were handed out by the general practice, patients may have been reluctant to rate continuity of care negatively, even if that might have reflected their true perception.

## Conclusion

The Nijmegen Continuity Questionnaire is a valid instrument to capture continuity of care. Even though it has been widely used previously, actual evidence on applicability in health systems outside of the Netherlands is scarce. Our study showed that, overall, a German translation of the NCQ in its current form yields results comparable to the original version and hence can be successfully applied to the German health care context. Still, compared to the Netherlands, parts of the latent constructs are higher correlated with each other and some items could benefit from a more precise wording, as they may be understood ambiguously by respondents and could be a potential source of bias.

## Supplementary Information

**Additional file 1.** German translation of the Nijmegen Continuity Questionnaire used in the study.

**Additional file 2.** Sociodemographic characteristics of patients participating in the quantitative study (additional subsamples, total sample for comparison).

**Additional file 3.** Detailed breakdown of missing values per item in the Nijmegen Continuity Questionnaire.

## Data Availability

The dataset supporting the conclusions of this article is not publicly available due to ongoing research. However, it is available for research purposes from the corresponding author upon reasonable request.

## Abbreviations

CFA: Confirmatory factor analysis
CFI: Comparative fit index
COREQ: Consolidated Criteria for Reporting Qualitative Studies
DMP: Disease Management Programme
EFA: Exploratory factor analysis
GP: General practitioner
N: Number of cases
NCQ: Nijmegen Continuity Questionnaire
RMSEA: Root Mean Square Error of Approximation
SD: Standard deviation
SRMR: Standardized Root Mean Square Residual
STROBE: Strengthening the reporting of observational studies in epidemiology
TLI: Tucker-Lewis-Index

## References

[1] Roth GA, Abate D, Abate KH, Abay SM, Abbafati C, Abbasi N (2018). Global, regional, and national age-sex-specific mortality for 282 causes of death in 195 countries and territories, 1980–2017: a systematic analysis for the Global Burden of Disease Study 2017. The Lancet.

[2] Amelung V, Hildebrandt H, Wolf S (2012). Integrated care in Germany-a stony but necessary road!. Int J Integr Care.

[3] Busse R, Blümel M, Knieps F, Bärnighausen T (2017). Statutory health insurance in Germany: a health system shaped by 135 years of solidarity, self-governance, and competition. The Lancet.

[4] Salisbury C, Sampson F, Ridd M, Montgomery AA (2009). How should continuity of care in primary health care be assessed?. Br J Gen Pract.

[5] Bice TW, Boxerman SB (1977). A quantitative measure of continuity of care. Med Care.

[6] Breslau N, Reeb KG (1975). Continuity of care in a university-based practice. J Med Educ.

[7] Uijen AA, Schellevis FG, van den Bosch WJ, Mokkink HG, van Weel C, Schers HJ (2011). Nijmegen Continuity Questionnaire: development and testing of a questionnaire that measures continuity of care. J Clin Epidemiol.

[8] Uijen AA, Schers HJ, Schellevis FG, Mokkink HG, van Weel C, van den Bosch WJ (2012). Measuring continuity of care: psychometric properties of the Nijmegen Continuity Questionnaire. Br J Gen Pract.

[9] Qiu C, Chen S, Yao Y, Zhao Y, Xin Y, Zang X (2019). Adaption and validation of Nijmegen continuity questionnaire to recognize the influencing factors of continuity of care for hypertensive patients in China. BMC Health Serv Res.

[10] Hetlevik O, Hustoft M, Uijen A, Assmus J, Gjesdal S (2017). Patient perspectives on continuity of care: adaption and preliminary psychometric assessment of a Norwegian version of the Nijmegen Continuity Questionnaire (NCQ-N). BMC Health Serv Res.

[11] Cohen Castel O, Dagan E, Keinan-Boker L, Shadmi E. Reliability and validity of the Hebrew version of the Nijmegen Continuity Questionnaire for measuring patients’ perceived continuity of care in oral anticancer therapy. Eur J Cancer Care (Engl). 2018;27(6):e12913. 10.1111/ecc.12913 30238665

[12] Arnold C, Hennrich P, Koetsenruijter J, van Lieshout J, Peters-Klimm F, Wensing M (2020). Cooperation networks of ambulatory health care providers: exploration of mechanisms that influence coordination and uptake of recommended cardiovascular care (ExKoCare): a mixed-methods study protocol. BMC Fam Pract.

[13] von Elm E, Altman DG, Egger M, Pocock SJ, Gøtzsche PC, Vandenbroucke JP (2007). The Strengthening the Reporting of Observational Studies in Epidemiology (STROBE) statement: guidelines for reporting observational studies. The Lancet.

[14] Tong A, Sainsbury P, Craig J (2007). Consolidated criteria for reporting qualitative research (COREQ): a 32-item checklist for interviews and focus groups. Int J Qual Health Care.

[15] Hu LT, Bentler PM (1999). Cutoff criteria for fit indexes in covariance structure analysis: Conventional criteria versus new alternatives. Struct Equ Model: A Multidisciplinary J.

[16] R Core Team (2021). R: A language and environment for statistical computing.

[17] Niemand T, Mai R (2018). Flexible cutoff values for fit indices in the evaluation of structural equation models. J Acad Mark Sci.

[18] Wensing M, Szecsenyi J, Laux G (2021). Continuity in general practice and hospitalization patterns: an observational study. BMC Fam Pract.

[19] Chyung SYY, Roberts K, Swanson I, Hankinson A (2017). Evidence-Based Survey Design: The Use of a Midpoint on the Likert Scale. Perform Improv.

[20] Krosnick JA, Alwin DF (1987). An Evaluation of a Cognitive Theory of Response-Order Effects in Survey Measurement. Public Opin Quart.

