# Supplementary material for: Measuring continuity of ambulatory cardiovascular care: a cross-sectional study on the applicability of the Nijmegen Continuity Questionnaire in Germany
Source: BMC Health Serv Res. 2022 Oct 18;22:1258. doi: 10.1186/s12913-022-08612-z (PMC9578194; doi:10.1186/s12913-022-08612-z)
Supplement: Supplementary file 1 — Additional file 1. German translation of the Nijmegen Continuity Questionnaire used in the study. [file 12913_2022_8612_MOESM1_ESM.docx]

Additional file 1: German translation of the Nijmegen Continuity Questionnaire that was used in the study (English version added afterwards).

Teil 3: Ihre Gesundheitsversorgung

Wir sind an Ihren Erfahrungen und Eindrücken bezüglich Ihrer Gesundheitsversorgung in den letzten 12 Monaten interessiert. Bitte kreuzen Sie bei jeder der folgenden Aussagen die Antwort an, die am besten Ihrer Meinung entspricht.

| Die folgenden Aussagen beziehen sich auf Ihren eigenen Hausarzt  Der eigene Hausarzt ist derjenige Arzt, den Sie bei einer Erkrankung in der Regel als erstes aufsuchen.  Wenn Sie Ihren Hausarzt in den letzten 12 Monaten nicht gesehen haben, fahren Sie bitte mit Frage 3.9 fort. | | | | | | |
| --- | --- | --- | --- | --- | --- | --- |
|  | Stimmt völlig | Stimmt | Neutral | Stimmt nicht | Stimmt gar nicht | Weiß nicht/ Unklar |
| 3.1 Ich kenne meinen Hausarzt sehr gut. |  |  |  |  |  |  |
| 3.2 Mein Hausarzt kennt meine Krankengeschichte sehr gut. |  |  |  |  |  |  |
| 3.3 Mein Hausarzt weiß immer sehr gut, was er/sie zuvor getan hat. |  |  |  |  |  |  |
| 3.4 Mein Hausarzt kennt meine familiären Umstände sehr gut. |  |  |  |  |  |  |
| 3.5 Mein Hausarzt kennt meine täglichen Aktivitäten sehr gut |  |  |  |  |  |  |
| 3.6 Mein Hausarzt kontaktiert mich, wenn nötig, ich muss ihn nicht darum bitten. |  |  |  |  |  |  |
| 3.7 Mein Hausarzt weiß sehr gut, was ich bei meiner Versorgung als wichtig erachte. |  |  |  |  |  |  |
| 3.8 Mein Hausarzt hält ausreichend Kontakt mit mir, wenn ich von anderen medizinischen Leistungserbringern (z.B. Physiotherapeut) betreut werde. |  |  |  |  |  |  |

| Die folgenden Aussagen beziehen sich auf die Kooperation zwischen Leistungserbringern in der Hausarztpraxis (z.B. zwischen dem Hausarzt und der Arzthelferin/Sprechstundenhilfe oder zwischen verschiedenen Hausärzten)  Wenn dieser Abschnitt nicht auf Sie zutrifft, fahren Sie bitte mit Frage 3.13 fort. | | | | | | |
| --- | --- | --- | --- | --- | --- | --- |
|  | Stimmt völlig | Stimmt | Neutral | Stimmt nicht | Stimmt gar nicht | Weiß nicht/Unklar |
| 3.9 Diese Leistungserbringer geben Informationen sehr gut aneinander weiter. |  |  |  |  |  |  |
| 3.10 Diese Leistungserbringer arbeiten sehr gut zusammen. |  |  |  |  |  |  |
| 3.11 Die Versorgung durch diese Leistungserbringer ist gut verbunden. |  |  |  |  |  |  |
| 3.12 Diese Leistungserbringer wissen immer sehr gut, was die anderen jeweils tun. |  |  |  |  |  |  |

| Die folgenden Aussagen beziehen sich auf Ihren eigenen Kardiologen  Der eigene Kardiologe ist der Herzspezialist, der sich überwiegend um die Behandlung ihrer Herz-/Kreislauferkrankung kümmert.  Wenn Sie in den letzten 12 Monaten keinen Kardiologen gesehen haben, fahren Sie bitte mit Frage 3.21 fort. | | | | | | |
| --- | --- | --- | --- | --- | --- | --- |
|  | Stimmt völlig | Stimmt | Neutral | Stimmt nicht | Stimmt gar nicht | Weiß nicht/ Unklar |
| 3.13 Ich kenne diesen Kardiologen sehr gut. |  |  |  |  |  |  |
| 3.14 Dieser Kardiologe kennt meine Krankengeschichte sehr gut. |  |  |  |  |  |  |
| 3.15 Dieser Kardiologe weiß immer sehr gut, was er/sie zuvor getan hat. |  |  |  |  |  |  |
| 3.16 Dieser Kardiologe kennt meine familiären Umstände sehr gut. |  |  |  |  |  |  |
| 3.17 Dieser Kardiologe kennt meine täglichen Aktivitäten sehr gut. |  |  |  |  |  |  |
| 3.18 Dieser Kardiologe kontaktiert mich, wenn nötig, ich muss nicht darum bitten. |  |  |  |  |  |  |
| 3.19 Dieser Kardiologe weiß sehr gut, was ich bei meiner Versorgung als richtig erachte. |  |  |  |  |  |  |
| 3.20 Dieser Kardiologe hält ausreichend Kontakt mit mir, wenn ich von anderen medizinischen Leistungserbringern betreut werde. |  |  |  |  |  |  |

| Die folgenden Aussagen beziehen sich auf die Kooperation zwischen Ihrem Hausarzt und Ihrem Kardiologen.  *Wenn dieser Abschnitt nicht auf Sie zutrifft, fahren Sie bitte mit Frage 4.1 fort.* | | | | | | |
| --- | --- | --- | --- | --- | --- | --- |
|  | Stimmt völlig | Stimmt | Neutral | Stimmt nicht | Stimmt gar nicht | Weiß nicht/ Unklar |
| 3.21 Diese Leistungserbringer geben Informationen sehr gut aneinander weiter. |  |  |  |  |  |  |
| 3.22 Diese Leistungserbringer arbeiten sehr gut zusammen. |  |  |  |  |  |  |
| 3.23 Die Versorgung durch diese Leistungserbringer ist sehr gut verbunden. |  |  |  |  |  |  |
| 3.24 Diese Leistungserbringer wissen immer sehr gut, was die anderen Leistungserbringer getan haben. |  |  |  |  |  |  |

English version, adapted from: Uijen AA, Schers HJ, Schellevis FG, Mokkink HG, van Weel C, van den Bosch WJ. Measuring continuity of care: psychometric properties of the Nijmegen Continuity Questionnaire. Br J Gen Pract. 2012;62(600):e949-57.

Part 3: Health care

We are interested in your experiences and impressions regarding health care in the past 12 months. For each statement, please indicate the answer that suits your opinion best.

| The following statements refer to your own general practitioner (GP)  Your own GP is the physician that you typically consult at first in case of a disease.  If you did not see your GP in the past 12 months, please continue with question 3.9. | | | | | | |
| --- | --- | --- | --- | --- | --- | --- |
|  | Completely correct | Correct | Neutral | Incorrect | Completely incorrect | Don’t know/ not sure |
| 3.1 I know my GP very well. |  |  |  |  |  |  |
| 3.2 My GP knows my medical history very well. |  |  |  |  |  |  |
| 3.3 My general practitioner always remembers, what he/she did on my last visit. |  |  |  |  |  |  |
| 3.4 My GP knows my familial circumstances very well. |  |  |  |  |  |  |
| 3.5 My GP knows very well what I do in my everyday life. |  |  |  |  |  |  |
| 3.6 If necessary, my GP gets in touch with me without me having to ask him/her for it. |  |  |  |  |  |  |
| 3.7 My GP knows very well what I deem important when it comes to my treatment. |  |  |  |  |  |  |
| 3.8 My GP keeps in touch sufficiently when I’m treated by other medical care providers (e.g. physiotherapists). |  |  |  |  |  |  |

| The following statements refer to the cooperation between care providers in your GP’s practice (e.g. between the GP and the physician’s assistant or between several GPs)  If this section does not apply to you, please continue with question 3.13. | | | | | | |
| --- | --- | --- | --- | --- | --- | --- |
|  | Completely correct | Correct | Neutral | Incorrect | Completely incorrect | Don’t know/ not sure |
| 3.9 These care providers exchange information very well. |  |  |  |  |  |  |
| 3.10 These care providers cooperate very well. |  |  |  |  |  |  |
| 3.11 Care by the care providers is linked together very well. |  |  |  |  |  |  |
| 3.12 The care providers always know very well, what the other care providers have done. |  |  |  |  |  |  |

| The following statements refer to your own cardiologist.  Your own cardiologist is the one who mainly deals with the treatment of your heart/circulation diseases.  If you did not see a cardiology within the past 12 months, please continue with question 3.21. | | | | | | |
| --- | --- | --- | --- | --- | --- | --- |
|  | Completely correct | Correct | Neutral | Incorrect | Completely incorrect | Don’t know/ not sure |
| 3.13 I know this cardiologist very well. |  |  |  |  |  |  |
| 3.14 My cardiologist knows my medical history very well. |  |  |  |  |  |  |
| 3.15 My cardiologist always remembers, what he/she did on my last visit. |  |  |  |  |  |  |
| 3.16 My cardiologist knows my familial circumstances very well. |  |  |  |  |  |  |
| 3.17 My cardiologist knows very well what I do in my everyday life. |  |  |  |  |  |  |
| 3.18 If necessary, my cardiologist gets in touch with me without me having to ask him/her for it. |  |  |  |  |  |  |
| 3.19 My cardiologist knows very well what I deem important regarding my care. |  |  |  |  |  |  |
| 3.20 This cardiologist stays in touch sufficiently when it comes to my treatment. |  |  |  |  |  |  |

| The following statements refer to the cooperation between your GP and your cardiologist.  If this section does not apply to you, please continue with question 4.1. | | | | | | |
| --- | --- | --- | --- | --- | --- | --- |
|  | Completely correct | Correct | Neutral | Incorrect | Completely incorrect | Don’t know/ not sure |
| 3.21 These care providers exchange information very well. |  |  |  |  |  |  |
| 3.22 These care providers cooperate very well. |  |  |  |  |  |  |
| 3.23 Care by the care providers is linked together very well. |  |  |  |  |  |  |
| 3.24 These care providers always know very well, what the other care providers have done. |  |  |  |  |  |  |
