# Supplementary material for: Measuring continuity of ambulatory cardiovascular care: a cross-sectional study on the applicability of the Nijmegen Continuity Questionnaire in Germany
Source: BMC Health Serv Res. 2022 Oct 18;22:1258. doi: 10.1186/s12913-022-08612-z (PMC9578194; doi:10.1186/s12913-022-08612-z)
Supplement: Supplementary file 3 — Additional file 3. Detailed breakdown of missing values per item in the Nijmegen Continuity Questionnaire. [file 12913_2022_8612_MOESM3_ESM.docx]

**Detailed breakdown of missing values per item in the Nijmegen Continuity Questionnaire**

| **Item no.** | **Item name** | **Missing values on questions regarding general practitioner*** | | | **Missing values on questions regarding cardiologist** | | | **Missing values on questions regarding general practitioner and cardiologist**** | | |
| --- | --- | --- | --- | --- | --- | --- | --- | --- | --- | --- |
|  |  | Item not answered | Item answered ambiguously (more than one box was marked) | “I don’t know/not sure”-option used | Item not answered | Item answered ambiguously (more than one box was marked) | “I don’t know/not sure”-option used | Item not answered | Item answered ambiguously (more than one box was marked) | “I don’t know/not sure”-option used |
| 1 | I know my general practitioner/this cardiologist very well | 7 | 0 | 2 | 92 | 0 | 5 | n.a. | | |
| 2 | My general practitioner/this cardiologist knows my medical history very well | 12 | 0 | 3 | 94 | 0 | 6 | n.a. | | |
| 3 | My general practitioner/this cardiologist always knows very well what he/she did previously | 18 | 0 | 7 | 96 | 0 | 17 | n.a. | | |
| 4 | My general practitioner/this cardiologist knows my familial circumstances very well | 9 | 1 | 10 | 97 | 0 | 18 | n.a. | | |
| 5 | My general practitioner/this cardiologist knows my daily activities very well | 19 | 3 | 19 | 97 | 0 | 14 | n.a. | | |
| 6 | My general practitioner/this cardiologist contacts me if it is needed, I do not have to ask | 10 | 2 | 9 | 95 | 0 | 19 | n.a. | | |
| 7 | My general practitioner/this cardiologist knows very well what I believe is important in my care | 12 | 0 | 13 | 98 | 0 | 19 | n.a. | | |
| 8 | My general practitioner/this cardiologist keeps in contact sufficiently when I see other care providers | 12 | 0 | 16 | 99 | 0 | 30 | n.a. | | |
| 9 | These care providers transfer information very well to each other | 56 | 0 | 20 | n.a. | | | 84 | 2 | 31 |
| 10 | These care providers work together very well | 58 | 1 | 24 | n.a. | | | 83 | 1 | 40 |
| 11 | The care of these care providers is very well connected | 57 | 0 | 31 | n.a. | | | 87 | 1 | 42 |
| 12 | These care providers always know very well from each other what they do | 59 | 0 | 35 | n.a. | | | 86 | 1 | 52 |
| *Items 9-12 on cooperation refer to cooperation between providers within the general practice  **Items 9-12 on cooperation refer to cooperation between the general practitioner and the cardiologist | | | | | | | | | | |
